# Neural basis for behavioral plasticity during the parental life-stage transition in mice

**DOI:** 10.3389/fncir.2023.1340497

**Published:** 2024-01-16

**Authors:** Kazunari Miyamichi

**Affiliations:** RIKEN Center for Biosystems Dynamics Research, Kobe, Hyogo, Japan

**Keywords:** hypothalamus, preoptic area, oxytocin, paternal behaviors, estrogen

## Abstract

Parental care plays a crucial role in the physical and mental well-being of mammalian offspring. Although sexually naïve male mice, as well as certain strains of female mice, display aggression toward pups, they exhibit heightened parental caregiving behaviors as they approach the time of anticipating their offspring. In this Mini Review, I provide a concise overview of the current understanding of distinct limbic neural types and their circuits governing both aggressive and caregiving behaviors toward infant mice. Subsequently, I delve into recent advancements in the understanding of the molecular, cellular, and neural circuit mechanisms that regulate behavioral plasticity during the transition to parenthood, with a specific focus on the sex steroid hormone estrogen and neural hormone oxytocin. Additionally, I explore potential sex-related differences and highlight some critical unanswered questions that warrant further investigation.

## Introduction

The adult brain possesses neuroplasticity, enabling it to adapt behaviors in response to specific life-stage demands. For instance, male laboratory mice, and certain strains of female mice, may exhibit aggressive behaviors leading to the killing of pups. Infanticide is thought to confer an evolutionary advantage by reducing potential competition for limited resources and thereby enhancing the survival prospects of the offender’s offspring (Lukas and Huchard, 2019). Infanticide can also expedite the mating of males with the mothers of the victim (Lukas and Huchard, 2014), as the reproductive cycle is typically suppressed during lactation. However, as the time approaches when animals anticipate their offspring, infanticide is suppressed and caregiving behaviors toward infants are greatly facilitated (Elwood, 1994; Dulac et al., 2014). In rodents, caregiving behaviors include nest building, retrieving scattered pups to a nest for protection from environmental hazards, and crouching over them for thermoregulation. Among these behaviors, pup retrieval has been widely used as a quantitative hallmark of parental behaviors (Yoshihara et al., 2018). Decades of research in rodents have indicated that aggressive and caregiving behaviors toward pups are regulated by distinct limbic neural types and circuits.

## Limbic neurons responsible for either parental or infanticidal behavior

**Figure 1:**
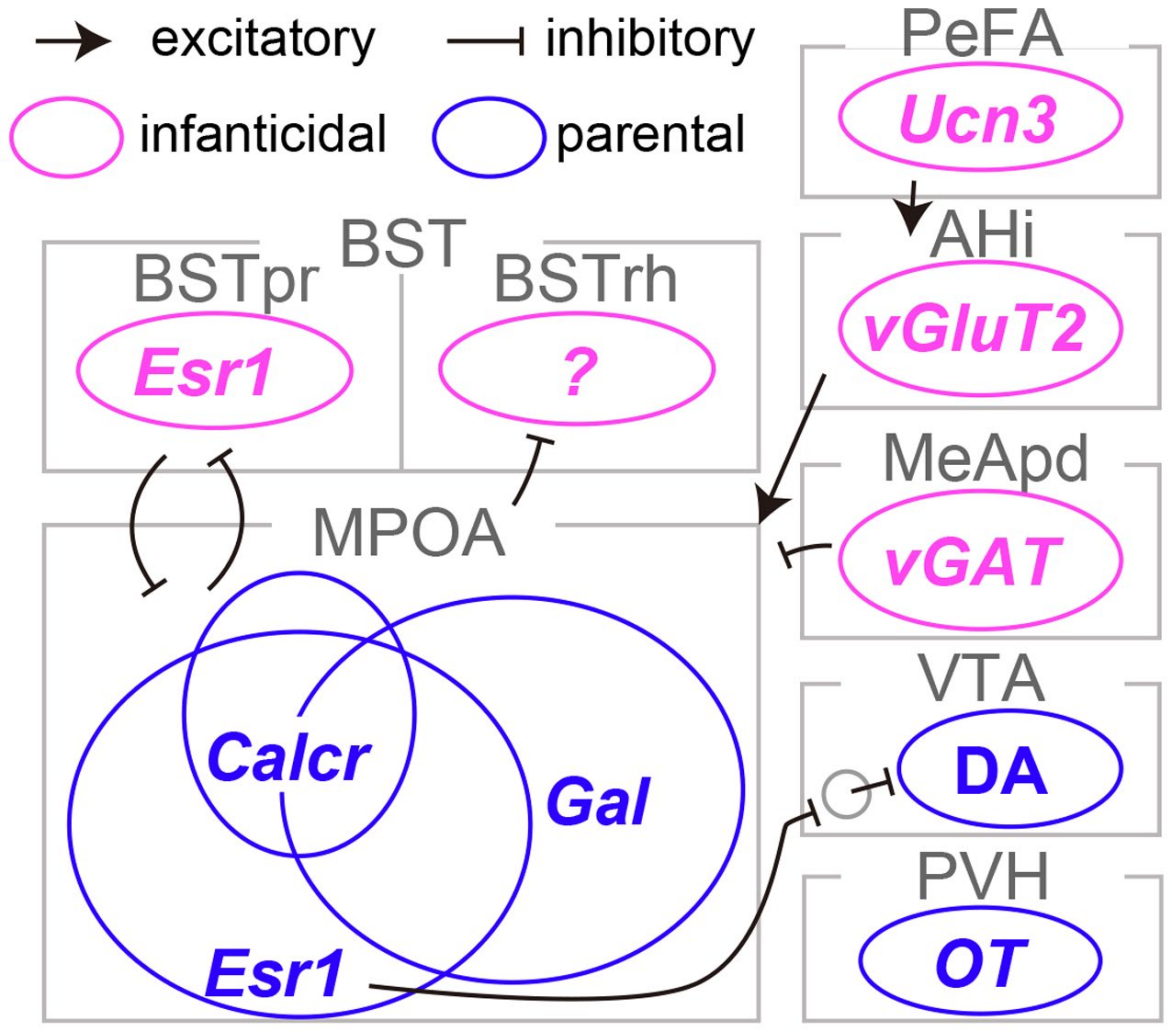
Limbic neurons responsible for infanticide and parental behaviors in mice. The distinct neural subtypes accountable for infanticide are depicted in magenta, whereas those fostering parental behaviors are illustrated in blue. Refer to the text for abbreviations and detailed information on connectivity.

The pioneering work of Numan (1974) established the critical role of the medial preoptic area (MPOA) in maternal behaviors in rats. Subsequent research has underscored the importance of the MPOA, particularly its central subdivision, in driving both maternal and paternal caregiving behaviors in mice (Tsuneoka et al., 2013). Wu et al. (2014) conducted a detailed examination of the MPOA at the level of molecularly defined cell types. This work led to the identification of *galanin*-expressing neurons (MPOA^Gal^ neurons), which showed frequent c-Fos expression, a proxy for neural activation, following maternal behaviors. Ablation of MPOA^Gal^ neurons resulted in the severe impairment of parental behaviors in both sexes. Conversely, optogenetic stimulation of these neurons in sexually naïve males effectively suppressed infanticidal behavior. Subsequent research by Kohl et al. (2018) employed rabies-virus-mediated transsynaptic tracing (Miyamichi et al., 2013) and its derivative method known as cTRIO (Schwarz et al., 2015) to dissect the input and output neural circuits associated with MPOA^Gal^ neurons. The findings revealed extensive reciprocal connectivity between MPOA^Gal^ neurons and various limbic structures, including those involved in transmitting pheromone signals originating from the vomeronasal organ, secreting neural hormones such as oxytocin and vasopressin, and mediating monoaminergic signals such as dopamine. Moreover, it was found that MPOA^Gal^ neurons consist of several distinct projection types that target different brain regions, such as the ventral tegmental area (VTA) and the medial amygdala (MeA). These MPOA^Gal^ neuron subtypes receive quantitatively varying inputs and may play a specific role in different aspects of parental behaviors, including pup-directed motor actions, the motivation for parental behaviors, and the inhibition of social intersections with adult conspecifics. Therefore, MPOA^Gal^ neurons act as a hub of parental behavioral regulation in both male and female mice (Figure 1).

Earlier research suggested the maternal behavior-facilitating effects of an estrogen surge during pregnancy on the function of the MPOA in female rats (Siegel and Rosenblatt, 1975). Inspired by this line of work, two independent studies demonstrated the pivotal role of MPOA neurons expressing estrogen receptor type 1 (MPOA^Esr1^ neurons) in the initiation and maintenance of maternal caregiving behaviors in mice (Fang et al., 2018; Wei et al., 2018). Specifically, MPOA^Esr1^ neurons were primarily GABAergic, exhibited heightened activity during the approach to pups and the initiation of pup retrieval, and induced pup retrieval when activated optogenetically (Fang et al., 2018). MPOA^Esr1^ neurons predominantly projected to non-dopaminergic (likely GABAergic) neurons within the VTA and enhanced maternal behaviors, presumably through the disinhibition of dopaminergic VTA neurons (VAT^DA^ neurons; Fang et al., 2018). Consistent with this perspective, VTA^DA^ neurons have been shown to display transient activity during pup retrieval and to be capable of encoding signals related to social rewards, thereby facilitating the efficient learning of pup retrieval behaviors in female mice (Xie et al., 2023). The acute silencing of VTA^DA^ neurons during pup retrieval results in a significant delay in the execution of these behaviors, reflecting the cumulative history of VTA^DA^ neuron activity. These lines of evidence lend support to the role of the MPOA^Esr1^ → VTA^DA^ circuity in maternal caregiving behaviors in mice (Figure 1); however, the function of this pathway in male mice remains elusive.

The MPOA plays a critical role in both parenting and sexual behaviors, as well as in the regulation of essential physiological functions such as body temperature control, thirst, and sleep (Zimmerman et al., 2017; Tan and Knight, 2018; Tsuneoka and Funato, 2021). The neural circuits governing these functions are likely to be composed of neurons with distinct genetic identities. Moffitt et al. (2018) utilized single-cell RNA sequencing and multiplexed error-robust fluorescence *in situ* hybridization to reveal that MPOA^Gal^ and MPOA^Esr1^ neurons overlapped and that each heterogeneous population encompassed a dozen transcriptome types. By combining cell-type classification and the detection of the *c-Fos* transcript, they suggested that a *calcitonin receptor* (*Calcr*) expressing MPOA neurons (MPOA^Calcr^ neurons) were predominantly active during parental behaviors in both males and females. Approximately 70% of MPOA^Calcr^ neurons overlap with MPOA^Esr1^ neurons, and the silencing of MPOA^Calcr^ neurons impairs maternal caregiving behaviors, whereas the chemogenetic activation of these neurons can suppress infanticide in sexually naïve male mice (Yoshihara et al., 2021). Additionally, when the *Calcr* gene was selectively suppressed in the MPOA, it resulted in partial impairment of maternal behaviors in risky environments. Collectively, MPOA^Calcr^ neurons represent the most clearly defined population for parental behaviors to date (Figure 1). It is worth noting that the potential relationship between the projection-based classification of MPOA^Gal^ neurons (Kohl et al., 2018) and their transcriptome types (Moffitt et al., 2018) remains uncertain and thus a subject for future investigation.

Olfactory signals play a critical role in pup-directed behaviors in rodents. The surgical or genetic elimination of the function of the vomeronasal organ, which is responsible for the detection of pheromonal signals in mice, has been shown to reduce infanticide in sexually naïve males, suggesting that the vomeronasal signals facilitate infanticide (Tachikawa et al., 2013; Isogai et al., 2018). Among the brain regions transmitting pheromonal signals, Tsuneoka et al. (2015) ascertained that *c-Fos* expression in the rhomboid nucleus of the bed nuclei of the stria terminalis (BSTrh) precisely reflected infanticidal motivation. Lesions in the BSTrh have been reported to inhibit infanticide in sexually naïve male mice. Subsequently, Chen et al. (2019) documented that *vGAT*-expressing GABAergic neurons in the medial amygdala posteroventral subdivision (MeApd^vGAT^ neurons) could elicit infanticidal behaviors in male mice, but not in females. Autry et al. (2021) reported that *urocortin-3* (*Ucn3*)-expressing neurons in the hypothalamic perifornical area (PeFA^Ucn3^ neurons) became active during attacks on infants in both males and females. These neurons received input from brain regions associated with pheromonal signals and stress. Functional manipulations of PeFA^Ucn3^ neurons have established their role in facilitating infanticide in both sexes, with notably vigorous attacks occurring when axonal projections of PeFA^Ucn3^ neurons to the amygdalohippocampal area (AHi) are optogenetically stimulated. Indeed, AHi contains excitatory projection neurons to the MPOA (AHi^→MPOA^ neurons) that exhibit activity in male mice during social interactions with pups and promote infanticide when chemogenetically activated (Sato et al., 2020). Furthermore, utilizing an outbred strain known as Rockland-Swiss mice, whose virgin females manifest a heightened propensity for infanticide, Mei et al. (2023) specifically examined the neural underpinnings of female infanticide. Their study disclosed that *Esr1*-expressing neurons in the principal nucleus of the BST (BSTpr^Esr1^ neurons) were imperative for the manifestation of, and could induce, infanticide in female mice. Taken together, these lines of evidence indicate the existence of a unique set of limbic neuron types that specifically regulate infanticidal behaviors in mice (Figure 1; Inada and Miyamichi, 2023).

## Hormonal regulations of parental behaviors in mice

How are infanticidal and parental behaviors appropriately regulated during the parental life-stage transition? Sex hormones exert a profound influence on reproductive and parental behaviors. Specifically, estrogen, a sex hormone responsible for the development and regulation of various reproductive functions, interacts with estrogen receptors, thereby modulating the expression of numerous genes (Knoedler et al., 2022). Maternal caregiving behaviors can be triggered by a substantial increase in estrogen and progesterone levels during pregnancy. As mentioned above, MPOA^Esr1^ neurons [which also express progesterone receptor (Pgr)] facilitate maternal behaviors. Do the steroid hormone receptors indeed function in these neurons? This question was addressed by Ammari et al. (2023) through their investigation of MPOA-specific conditional knockout (cKO) of *Esr1* or *Pgr*. Their study established essential roles of *Esr1* and *Pgr* in the pregnancy-induced enhancement of pup retrieval in expectant mother mice. Notably, substantial overlap was observed between MPOA^Gal^ and MPOA^Esr1^ neurons (Moffitt et al., 2018), and selective cKO of either *Esr1* or *Pgr* within the MPOA^Gal^ neurons reproduced the effects observed in pan-MPOA cKO mice. Thus, the proper expression of maternal behaviors in female mice requires the signaling of both estrogen and progesterone receptors within the MPOA^Esr1∧Gal^ neurons. Pregnancy induces substantial changes in the electrophysiological properties in an *Esr1*- and *Pgr*-dependent manner. During the late pregnancy period, MPOA^Gal^ neurons exhibit a long-lasting reduction in baseline activity and an increased level of excitability. At the individual cellular level, the representation of pup retrieval within MPOA^Gal^ neurons becomes sparser and more distinguishable from other non-pup-related signals, although whether this effect is mediated by *Esr1* or *Pgr* remains elusive. Taken together, these findings by Ammari et al. (2023) illustrated that sex steroid hormones reorganize the parental behavioral center, specifically the MPOA^Esr1∧Gal^ neurons, to enhance the efficient execution of parental behaviors during the maternal life-stage transition. Whether a similar mechanism plays a role during the paternal transition in male mice remains an open question.

As previously mentioned, an additional population expressing *Esr1*, namely the BSTpr^Esr1^ neurons, exert opposing control to trigger infanticide in female mice, which should be suppressed during the maternal transition. Notably, MPOA^Esr1^ and BSTpr^Esr1^ neurons communicate with each other via mutually inhibitory monosynaptic connections (Mei et al., 2023), as demonstrated through channelrhodopsin 2-assisted circuit mapping (CRACM; Petreanu et al., 2007). Terminal activation of BSTpr^Esr1^ neurons induces inhibitory postsynaptic currents in the majority of MPOA^Esr1^ neurons, and vice versa. These antagonistic connections hold functional significance, as optogenetic suppression of MPOA^Esr1^ → BSTpr^Esr1^ neuron connections leads to infanticide, whereas optogenetic activation of the same pathway inhibits infanticide. Similarly, virgin female mice display inhibited or activated infanticidal behaviors when BSTpr^Esr1^ → MPOA^Esr1^ neuron connections are optogenetically suppressed or activated, respectively. At the population level, BSTpr^Esr1^ neurons become active during hostile investigation and infanticidal episodes, whereas MPOA^Esr1^ neurons become active during pup retrieval. Upon the maternal life-stage transition, the excitabilities of MPOA^Esr1^ and BSTpr^Esr1^ neurons undergo substantial changes. In mothers, MPOA^Esr1^ neurons become more excitable, whereas BSTpr^Esr1^ neurons become significantly less excitable. The report by Mei et al. (2023) collectively illustrated the life-stage-associated alteration of excitability in antagonistic circuits that mediate infanticide and maternal care in female mice. The exact function of estrogen receptors in BSTpr^Esr1^ neurons remains uncertain and is thus a subject for future study.

In addition to steroid hormones, peptidergic hormones may contribute to the parental behavioral transition. Particularly, oxytocin (OT), a nonapeptide hormone produced by OT neurons in the paraventricular (PVH^OT^ neurons) and supraoptic (SO) hypothalamic nuclei, plays a pivotal role in regulating sexual, maternal, and social behaviors, in addition to its classical functions in uterine contractions during parturition and milk ejection during lactation (Nishimori et al., 1996; Macbeth et al., 2010; Froemke and Young, 2021; Yukinaga et al., 2022). Intracerebroventricular (Pedersen et al., 1982) and intraperitoneal (Marlin et al., 2015) administrations of OT have been shown to trigger caregiving behaviors in virgin rodent females, in addition to the optogenetic activation of PVH^OT^ neurons (Marlin et al., 2015; Scott et al., 2015). By contrast, loss-of-function of OT or its receptor, OTR, shows relatively minor phenotypes in maternal caregiving behaviors (Nishimori et al., 1996; Young et al., 1996; Macbeth et al., 2010), except in situations of food scarcity and high stress (Ragnauth et al., 2005). Brain region-specific cKO of the *OT* gene within the PVH and SO further corroborates its dispensability in maternal caregiving behaviors (Hagihara et al., 2023). Collectively, these studies suggest that OT signaling can facilitate the onset, but to a lesser extent, the maintenance of maternal care (Yoshihara et al., 2018).

The modulation of the sensory system stands out as a critical mechanism through which OT exerts its influence on maternal behaviors. For instance, the auditory system plays an important role in mediating the distinct vocalizations emitted by offspring to facilitate maternal behaviors. Cohen et al. (2011) reported the experience-dependent and pup-odor-induced alterations of neural responses within the mother’s primary auditory cortex, resulting in an elevated sensitivity to the pup’s ultrasonic vocalizations. The pairing of pup vocalization with OT administration produces enduring changes in neural responses, augmenting excitatory responses by adjusting the local excitatory/inhibitory (E/I) balance (Marlin et al., 2015; Schiavo et al., 2020). Virgin females can employ their visual system to acquire pup retrieval behaviors from experienced mother mice, during which time, the activation of PVH^OT^ neurons and concurrent modulation of auditory sensitivity occurs (Carcea et al., 2021). Furthermore, pup vocalization can elicit an enduring activation of PVH^OT^ neurons through a specific thalamic neural circuit (Valtcheva et al., 2023). These studies have collectively established connections between cellular and synaptic properties, the physiological impacts of OT, and the onset of pup retrieval. In addition, while the precise implications for maternal behaviors remain unclear, it is well established that OT can modulate various other sensory systems, including the olfactory cortex (Oettl et al., 2016).

In contrast to the relatively moderate modulatory roles of OT in female mice, Inada et al. (2022a) demonstrated that OT released from PVH^OT^ neurons is indispensable for paternal caregiving behaviors in male mice. They examined the PVH-specific cKO of the *OT* gene or the genetic removal of PVH^OT^ neurons, which resulted in a significant decrease in the number of pups retrieved and the duration of paternal care exhibited by male mice. The chemogenetic activation of PVH^OT^ neurons effectively suppresses infanticidal behaviors and, in turn, triggers caregiving behaviors in sexually naïve male mice, and this effect is dependent on *OT*. This activation of PVH^OT^ neurons heightens the activity of MPOA^Calcr^ neurons, which promote caregiving (Figure 1) while concurrently suppressing the activity of PeFA^Ucn3^ neurons, which promote infanticide. Another potential downstream target of PVH^OT^ neurons is the infanticide-promoting AHi^→MPOA^ neurons, as OT can suppress these neurons by facilitating local inhibitory neurons expressing OTRs (Sato et al., 2020). Therefore, PVH^OT^ neurons play a pivotal role in coordinating various limbic neural populations to favor the execution of parental behaviors in male mice. Notably, although the activity dynamics of PVH^OT^ neurons during paternal behaviors remain largely unknown in mice, biparental male mandarin voles display time-locked activities of PVH^OT^ neurons to each episode of paternal caregiving behaviors, such as pup retrieval and sniffing of pups (He et al., 2021).

**Figure 2:**
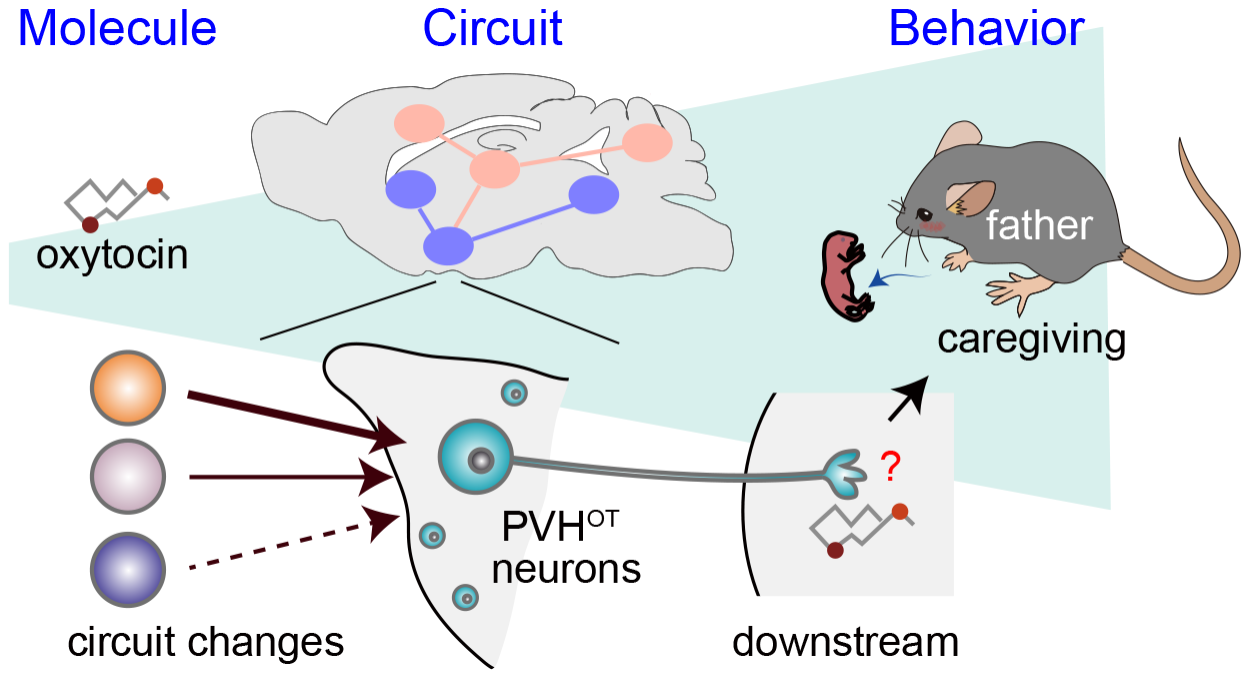
A circuit mechanism to facilitate paternal behaviors by PVH^OT^ neurons. Illustration depicting alterations in neural circuitry within the brains of paternal mice and the oxytocin-mediated facilitation of caregiving behavior (Inada et al., 2022a). The downstream neural processes associated with OT in promoting paternal behaviors remain unidentified, as denoted by the red question mark.

As a potential mechanism underlying the activation of PVH^OT^ neurons in father mice, Inada et al. (2022a) demonstrated that individual PVH^OT^ neurons in father mice receive a quantitatively greater amount of excitatory synaptic input from specific hypothalamic nuclei, including the lateral hypothalamus (LHA^vGluT2^ neurons). This insight was gained utilizing rabies virus-based transsynaptic tracing (Miyamichi et al., 2013) and CRACM (Petreanu et al., 2007). The heightened LHA^vGluT2^ → PVH^OT^ neuron connectivity appears to have functional significance, as chemogenetic activation of LHA^vGluT2^ neurons suppresses infanticide in a downstream PVH^OT^ neuron-dependent manner. Taken together, these findings suggest that the promotion of paternal caregiving behaviors hinges on structural plasticity within the hypothalamus of fathers, resulting in the increased excitability of PVH^OT^ neurons (Figure 2).

## Perspective

As outlined above, recent research in mice employing a viral-genetic approach has elucidated that estrogen and OT exert their effects on specific limbic neural types, thereby modulating circuit functions to suppress infanticide and promote parental behaviors. Notably, the extent to which females and males depend on estrogen and OT to trigger parental behaviors appears to differ. In female mice, both estrogen and OT can facilitate maternal behaviors, with estrogen playing a more pivotal role. This can account for the relatively modest reliance of female mice on OT for maternal caregiving behaviors *per se*, despite the critical role of OT for milk ejection (Hagihara et al., 2023). Conversely, the involvement of estrogen-dependent mechanisms in male mice remains uncertain, though it is unlikely to be indispensable (Wynne-Edwards and Timonin, 2007). Instead, they rely more significantly on OT to express paternal caregiving behaviors (Inada et al., 2022a).

While prior research has proposed that paternal caregiving behaviors may be facilitated by mating, cohabitation with a female, and/or repeated exposure to pups (vom Saal, 1985; Elwood, 1994; Cai et al., 2021), the specific triggers for the sufficient activation of OT neurons and their input structural plasticity remain open questions. Moreover, although OT-induced paternal behaviors are associated with the activation of MPOA^Calcr^ neurons, the mechanisms through which OT exerts its facilitatory effects on paternal behaviors, including brain regions and receptor mechanisms, require more elucidation (Figure 2). Related to this issue, it is worth noting that the OTR-based modulation of inhibitory neurons has been reported in both the primary auditory cortex (Marlin et al., 2015) and AHi (Sato et al., 2020). Furthermore, Esr1 appears to augment inhibitory tones within the MPOA, thereby differentiating the representation of pups in late pregnant females (Ammari et al., 2023). Modulation of the excitatory/inhibitory balance to enhance the saliency of pup-related signals may represent a common mechanism for facilitating parental behaviors by OT and Esr1.

More broadly, intricate patterns in the levels of hormone and receptor expression have been observed in classical studies involving biparental model rodents. However, the behavioral implications of these dynamic endocrinological changes remain largely uncertain (Wynne-Edwards and Timonin, 2007). To address this limitation, two research avenues should be pursued: (i) examining the functions of hormones and receptors in a stage- and cell-type-specific manner, as illustrated by recent cKO models targeting *Esr1*, *Prolactin receptor*, *OT, and OTR* (Stagkourakis et al., 2020; Inada et al., 2022a,b; Ammari et al., 2023); and (ii) employing fluorescent biosensors to characterize the high spatiotemporal hormonal dynamics during specific behavioral episodes, as exemplified by the heightened OT secretion from PVH^OT^ neurons during mating in male mice (Qian et al., 2023). Future studies that broaden the application of these techniques hold promise for unraveling the functions of each hormone at every stage of the parental life-stage transition.

Lastly, it is important to mention that the execution of parental behaviors is not solely the province of limbic neurons; it requires the coordinated function of multiple brain regions to process infant cues, make decisions, and formulate and execute motor plans. These processes likely demand higher cognitive functions, and researchers have only recently begun to explore these avenues (Corona et al., 2023). Given the substantial evolutionary expansion of the human frontal cortex, understanding the higher-order functions associated with parental behaviors and the potential interactions between the frontal cortex and limbic circuits during such behaviors are expected to offer valuable insights into human parental behaviors.

## Author contributions

The author confirms being the sole contributor of this work and has approved it for publication.
